# Obesity and type 2 diabetes as chronic inflammation: how does the cytokine evidence align?

**DOI:** 10.3389/fendo.2026.1721206

**Published:** 2026-02-03

**Authors:** Karishma Bhatia, Vikas Kumar Gupta, Sanjeev K. Upadhyay

**Affiliations:** Department of Biochemistry, Faculty of Science, The Maharaja Sayajirao University of Baroda, Vadodara, Gujarat, India

**Keywords:** adipokine, cytokine, diabetes type 2, inflammation, obesity

## Abstract

Obesity is a global epidemic and a major risk factor for several non-communicable diseases. Systemic inflammation is believed to be involved in obesity and obesity-induced diabetes type 2, which alters adipose tissue homeostasis. Cytokines, the key mediators of inflammation, play a central role in this inflammatory state and have been extensively studied for their role in obesity and diabetes type 2. Therefore, blood and adipose tissue levels of cytokines have been a subject of intense investigation over the last two decades. Several studies reveal the role of cytokines and their profiles in the obese population. These studies have reported the significance of altered levels and patterns of several cytokines and their association with clinical parameters in obese and type 2 diabetics. This review examines population-based studies to evaluate whether cytokine profile consistently reflect chronic inflammation in obesity and type 2 diabetes. It highlights cytokines that show robust associations across ethnic and geographic cohorts. While majority of cytokines are frequently elevated in both conditions, their predictive value remains unclear. On the contrary we do find inflammatory cytokines like IL-1β which shows an association with diabetes type 2 but not obesity while IL-6 is more closely associated with obesity than diabetes. Anti-inflammatory cytokines IL-10 and IL-4 cannot be linked to either conditions. The study underscores the need for longitudinal and mechanistic studies to determine whether cytokine profiling could be used as an early diagnostic or prognostic tool.

## Graphical Abstract

**Figure:**
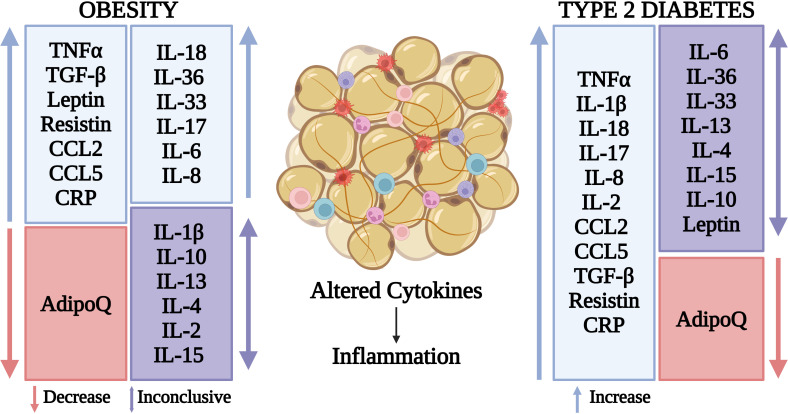

## 1 Introduction

Obesity has increased drastically in the last couple of decades. A Lancet report of 2024 puts the number of individuals with obesity at over one billion (1). Its incidence is higher among women than men and it is expected to affect half of adult population by mid-century. It is a major concern not only in the developed world but even in developing countries like India (2, 3). During the development of obesity, abnormal expansion of adipose tissues causes functional impairment often associated with lipotoxicity, hypoxia, metabolic inflammation and insulin resistance (IR) (4). Adipocyte lipid turnover is an essential factor in dyslipidemia (5). Free fatty acids (FFAs) released from adipocytes initiate unfolded protein response (UPR) via JNK activation and induce inflammation and oxidative stress that contributes to IR (6). The earliest studies in the 1960s showed that obesity is linked to the infiltration of immune cells in adipose tissue (7, 8). More than a decade later, macrophages were shown to affect the insulin action on adipocytes (9). These observations were further reinforced when the Spiegelman group showed elevated TNF-α in individuals with obesity and its relation to IR (10, 11). These studies laid the foundation for inflammation as a critical driver of obesity and IR. Increased adiposity in mice causes local hypoxia associated with increased T cell population within adipose tissue (12). Similarly, a hypoxic condition in human adipose tissue correlates with macrophage infiltration and cytokine secretion (13). However, in another study, men with obesity were found to have higher pO_2_ in the adipose tissue compared to lean subjects with impaired vascularization and higher levels of inflammation (14). This study hints at a possible lower metabolic rate of adipose tissue as a significant reason for IR. HIF-1α is the critical signaling molecule in hypoxia, leading to fibrosis, inflammation and metabolic dysfunction (15). HIF-1α redirects the adipose tissue macrophages (ATMs) metabolism towards glycolysis and altered mitochondrial function (16) that induces cytokine secretion (17). Fat accumulation in adipose tissue increases oxidative stress, altering adipokine levels via NOX upregulation (18). In addition, HIF-1α induction mounts a ‘fibrotic response’ by upregulation of collagen production, which causes adipose tissue fibrosis (19). The level of adipose tissue fibrosis also determines the benefits of gastric bypass surgeries in individuals with obesity (19). Interestingly, exercise delays fibrosis and improves IR (20, 21).

Metabolic stress and adipose tissue dysfunction are closely related to obesity. Inflammation in white adipose tissue has been recognized as a major contributor to systemic inflammation and disrupted insulin signaling (22, 23). Adipocytes and macrophages within the adipose tissue of individuals with obesity play a crucial role in producing inflammatory mediators, including monocyte chemoattractant protein-1 (MCP-1), tumor necrosis factor-alpha (TNF-α) and interleukin-6 (IL-6). These molecules further increase macrophage infiltration into the tissue. It has been proposed that adipokines such as leptin can induce T-helper cells to secrete pro-inflammatory cytokines like TNF-α and interferon-gamma (IFN-γ) (24). Besides T cells and macrophages, B-cells, dendritic cells, NK cells, neutrophils and mast cells also contribute to developing adipose tissue dysfunction (25). The primary language of communication between these players is cytokines, which becomes a measurable parameter to study inflammation. Cytokine profile is a diagnostic tool for many diseases (26, 27). Several clinical studies have studied the cytokine levels in subjects with obesity and diabetes type 2 (T2D). In this review, we are trying to summarize clinical results that can aid in a better understanding of pathophysiology of obesity and T2D and possible early diagnosis of the condition based on cytokine profile.

## 2 Cellular remodeling of adipose tissues during obesity

**Figure 1:**
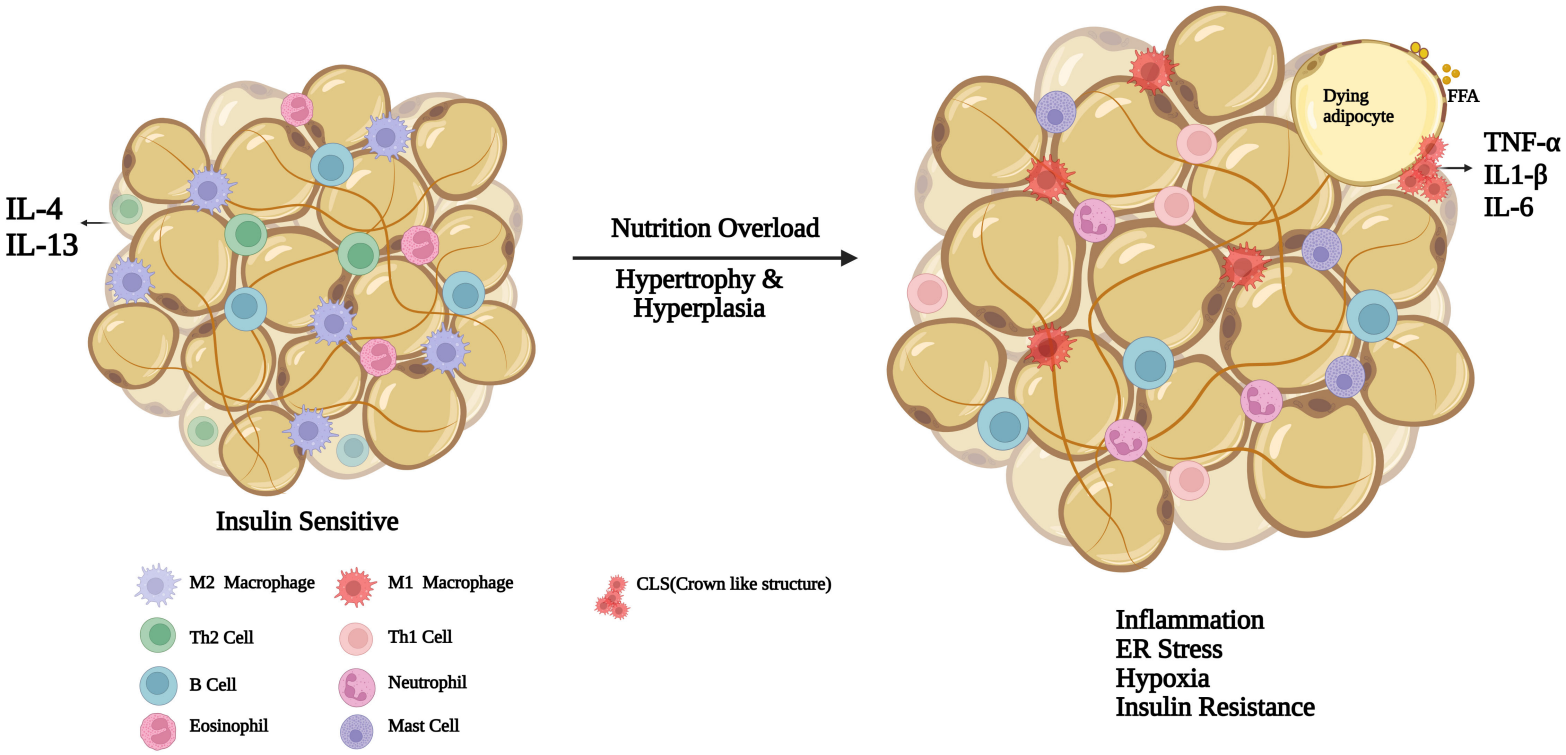
Immune cell distribution in lean versus obese adipose tissue: immune cell distribution and adipocyte changes in lean versus obese adipose tissue. In lean adipose tissue, adipocytes maintain a smaller size, with M2 macrophages dispersed throughout the tissue, promoting an anti-inflammatory, insulin-sensitive state. Lean adipose tissue contains regulatory immune cells, including Th2 cells and eosinophils, which secrete IL-4 and IL-13 to sustain anti-inflammatory signaling. In the obese adipose tissue, adipocytes undergo both hypertrophy and hyperplasia. Hypertrophic adipocytes are prone to rupture, releasing free fatty acids (FFAs), which attract and activate M1 macrophages that form crown-like structures (CLS) around damaged adipocytes. These M1 macrophages secrete proinflammatory cytokines. Eosinophils and regulatory Th2 decrease, while neutrophils, Th1, B cells and mast cells increase. The accumulation of proinflammatory immune cells and cytokines in the obese adipose tissue leads to chronic inflammation, contributing to endoplasmic reticulum (ER) stress and subsequent insulin resistance. Macrophages accumulate around apoptotic adipocytes to form Crown like Structures (CLS). Created in BioRender.

Adipose tissue plays a pivotal role in energy homeostasis. These are of two types. Brown adipose tissue (BAT) is involved in non-shivering thermogenesis, while white adipose tissue (WAT) stores excess calories and maintains metabolic equilibrium. A third type, called inducible BAT or beige adipocytes have also gained attention. These are thermogenic in response to external stimuli like cold, exercise, PPARγ agonists, B3-adrenergic receptor agonists, and tissue injury (28). In addition, adipose tissue is also a vital endocrine organ, secreting various adipokines. Other than adipocytes, resident immune and endothelial cells are primary sources of several adipokines (29). Obesity causes an increase in the number of adipocytes (hyperplasia) as well as volume (hypertrophy), leading to an increase in adipose mass (30). It also alters the immune cell composition in the adipose tissue (**Figure 1**). Macrophages are the most abundant immune cells during obesity, constituting 40-50% of all the adipose tissue cells in obese mice compared to 10% in lean mice (31). Cinti et al. demonstrated that hypertrophic adipocyte death encourages macrophage accumulation in the adipose tissue around necrotic adipocytes. These macrophages accumulate to clear up the debris and often form a syncytium. When observed under microscope after immunostaining it appears like a crown over an adipocyte. These crown-like structures are a hallmark of obesity. Majority of macrophages within adipose tissue reside within these crown like structures (32). Diet-induced obesity involves a phenotypic shift in ATMs from an anti-inflammatory phenotype (M2) characterized by increased expression of Ym1, arginase1 and IL-10 to a pro-inflammatory state (M1) which express iNOS and TNF-α (33). High-fat diet aggravates pro-inflammatory cytokine secretion from M1 macrophages by activating TLR4, and contribute to IR (34). However, it has been demonstrated that ATMs in the obese adipose tissue, although pro-inflammatory, are distinct from M1 and express CD36, ABCA1 and PLIN2 on their surface. These ATMs also display increased lysosomal activity and sometimes referred as metabolically activated macrophages (MMe) (35–37). These macrophages exhibit distinct metabolic activation marked by enhanced oxidative phosphorylation (OXPHOS) and glycolysis (38). These metabolic alterations in ATMs promote adipose tissue dysfunction by altering the adipocyte mitochondrial bioenergetics (39).

Like in many other tissues, neutrophils infiltrate adipose tissues at a very early stage of calorie abundance and initiate inflammation (40). Increased elastase secretion by neutrophils is considered an early event in the development of inflammation (41). Ablation of neutrophil-specific elastase in mice resulted in resistance to diet induced obesity, reduced IR, increased fatty acid oxidation in the liver and decreased infiltration of macrophages in adipose tissue (41, 42). The data on the role of eosinophils is not conclusive and there are conflicting reports about their significance in obesity and IR. Some reports find eosinophils beneficial (43, 44), while others detrimental (45). An increase in mast cell population in the adipose tissue is associated with obesity. Genetic deficiency and pharmacological stabilization of mast cells in mice showed reduced obesity and inflammation with improved glucose tolerance and energy expenditure (46). 15-deoxy-ΔPGJ2 (a prostaglandin) secreted from mast cells induce adipocyte differentiation via activation of PPARγ (47). Increased mast cell proportion in the adipose tissue of adults with metabolic syndrome is related to waist circumference, blood glucose levels, HOMA-IR, leptin, inflammation (IL-6, IL-1β), as well as circulating macrophages (48).

Dendritic cells (DCs) are professional antigen-presenting cells essential to adipose tissue inflammation. Racic et al. demonstrated that increased DCs induced by a high-fat diet in mice were associated with a crown-like structure and promoted macrophage accumulation in the adipose tissue (49). Cho et al. described CD45^+^CD64^-^CD11c^+^ adipose tissue DCs, distinct from CD45^+^CD64^+^ adipose tissue macrophages that predominate in subcutaneous adipose tissue (SAT) (50). DCs can induce Th17 differentiation within adipose tissue, contributing to inflammation along with neutrophils and macrophages (51, 52). Thus, dendritic cells are linked innately to adaptive immune responses in obesity. CD4^+^ cells can be ascribed to one of the Th1, Th2, Th17 or Treg cells. Among these, Th1 and Th17 are positively correlated while Th2 and Treg cells are negatively correlated with obesity (53). Excessive nutrition promotes Th17 differentiation and their numbers have been correlated with glycated hemoglobin (HbA1c) in diabetic patients with obesity (54). Th17 cells appear to impair the insulin receptor signaling pathway and decrease insulin sensitivity by promoting the secretion of IL-17 and IL-22, thereby contributing to metabolic dysfunction (55). As obesity progresses, the number of Treg cells decrease, accompanied by an elevated Th17/Treg ratio. Obese mice show diminished Treg cells in their adipose tissue that regulate the synthesis of inflammatory molecules and surface expression of GLUT4 in adipocytes (56). Treg cells maintain insulin sensitivity in WAT by reducing inflammation and secreting IL-10 (57). Likewise, CD8^+^ T cells increase and Treg cells decrease, while adipose tissue macrophages accumulate in adipose tissue (58). Mice fed a high-fat diet and lacking B cells exhibit lower body weight, improved glucose homeostasis and reduced systemic inflammation (59). B cells are shown to secrete pro-inflammatory cytokines in obese while anti-inflammatory in lean mice (60). These also act as regulators of T cell functioning and thus modulate inflammation. B cell-deficient high fat diet fed mice show increased Treg population, decreased production of inflammatory cytokines, and improved glucose tolerance (60). However, Nishimura et al. have suggested that usually existing Breg cells in the adipose tissue produce IL-10 and thus impede inflammation in diet-induced obese mice. Even Breg cell markers and IL-10 expressions are inversely associated with BMI in human subjects (61). Therefore, immune dysregulation in the adipose tissue of individuals with obesity results in chronic low-grade inflammation. Moreover, the regional differences in immune cell localization within the adipose tissue may be essential to adipose tissue homeostasis, which we do not understand very well (62, 63).

## 3 Alterations in Inflammatory profile during obesity and diabetes type 2

The inflammatory state originates largely from dysfunctional adipose tissue and altered immune cell activity, leading to the release of numerous cytokines that modulate insulin signaling and metabolic homeostasis. Several cytokines such as TNF-α, IL-6, IL-1β and others have been widely studied in this context. Their altered levels in obese and diabetic populations have led to the hypothesis that they are not just biomarkers of disease, but active participants in its pathogenesis. However, whether these cytokine changes are causative, merely correlative, or predictive remains unclear. Here, we examine data from clinical and population based studies across diverse geographic and ethnic backgrounds and evaluate the patterns of consistency or contradiction. Although these studies have different outcomes (**Table 1**), they underline the role of cytokines in obesity and T2D.

**Table 1:** List of human studies which have measured different cytokine levels either from blood or adipose tissue.

Cytokine	Key role in obesity/T2D	Obesity (references)	Type 2 Diabetes (references)
TNF-α	Pro-inflammatory; impairs insulin signaling	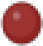 10, 67–77, 83–88 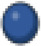 102–106	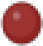 89, 91–95, 99 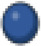 101, 106–108
IL-6	Pro-inflammatory; glucose metabolism; dual role	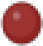 68, 71, 83–86, 114, 117– 124, 126, 127, 130 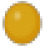 135 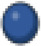 70, 88, 103, 105	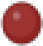 76, 94, 99, 108, 122, 129, 130 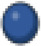 101
IL-1β	Promotes β-cell apoptosis; impairs insulin secretion	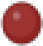 124, 144 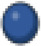 88, 99, 135, 145	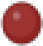 144, 149 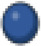 150
IL-18	Pro-inflammatory; caspase activation; energy homeostasis	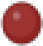 145, 155–157	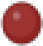 156, 157
IL-33	Immunomodulatory; regulates metabolism	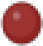 170, 171 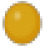 172	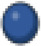 173
IL-36	Pro-inflammatory; induces cytokine production	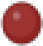 159, 160, 162	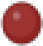 160
IL-17	Th17 derived, Pro-inflammatory; affects adipogenesis	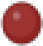 124, 140, 177, 178 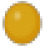 135	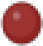 161
IL-10	Anti-inflammatory; enhances insulin sensitivity	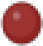 71, 72, 103, 104, 124 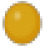 69, 93, 118, 187 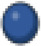 67, 100, 108 132,135	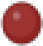 144 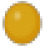 123, 131, 186, 188
IL-4	Anti-inflammatory; shifts to M2 macrophages, inhibits adipogenesis via STAT6	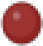 71, 101, 144 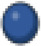 67,103, 132,135	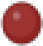 71, 144 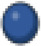 67
IL-13	Anti-inflammatory; regulates glucose metabolism	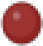 104 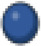 132	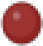 71, 93, 144 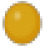 191
IL-2	Pro-inflammatory; modulates macrophage phenotype	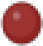 70, 124, 196 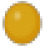 197 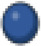 67, 103, 104, 132	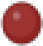 101
IL-15	Stimulates glucose uptake, reduces adiposity	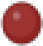 124 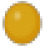 206, 207 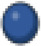 135	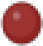 199, 201 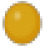 202
TGF-β	Pleiotropic; promotes inflammation, fibrosis	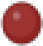 212-215	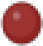 216, 217
Adiponectin	Anti-inflammatory; improves insulin sensitivity	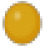 70, 72, 130, 197,207, 223, 226,231,232 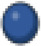 88, 105	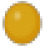 95, 96, 130, 228, 230 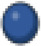 227
Leptin	Regulates energy balance and glucose homeostasis	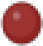 67, 74, 95, 129, 131, 177, 236, 238, 239	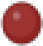 239 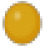 241-243
Resistin	Induces insulin resistance, inflammation	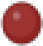 207, 252	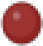 133, 252, 253
Chemokines	Chemotactic; promotes macrophage recruitment	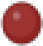 122, 125, 134, 155 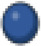 103, 135	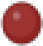 101
IL-8	Chemokine; inhibits Akt, induces insulin resistance	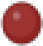 71, 121, 259 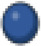 103, 117	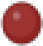 144
CRP	Marker of systemic inflammation	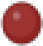 67, 68, 74, 99, 107, 122, 123, 129,130, 134, 145, 261 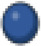 70, 135	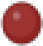 100, 130, 144, 206, 229, 263 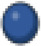 106, 125, 173

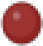
Increased; 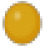
Decreased; 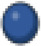
No significant change. Based on human studies measuring circulating or adipose tissue cytokine levels.

### 3.1 TNF-α

**Figure 2:**
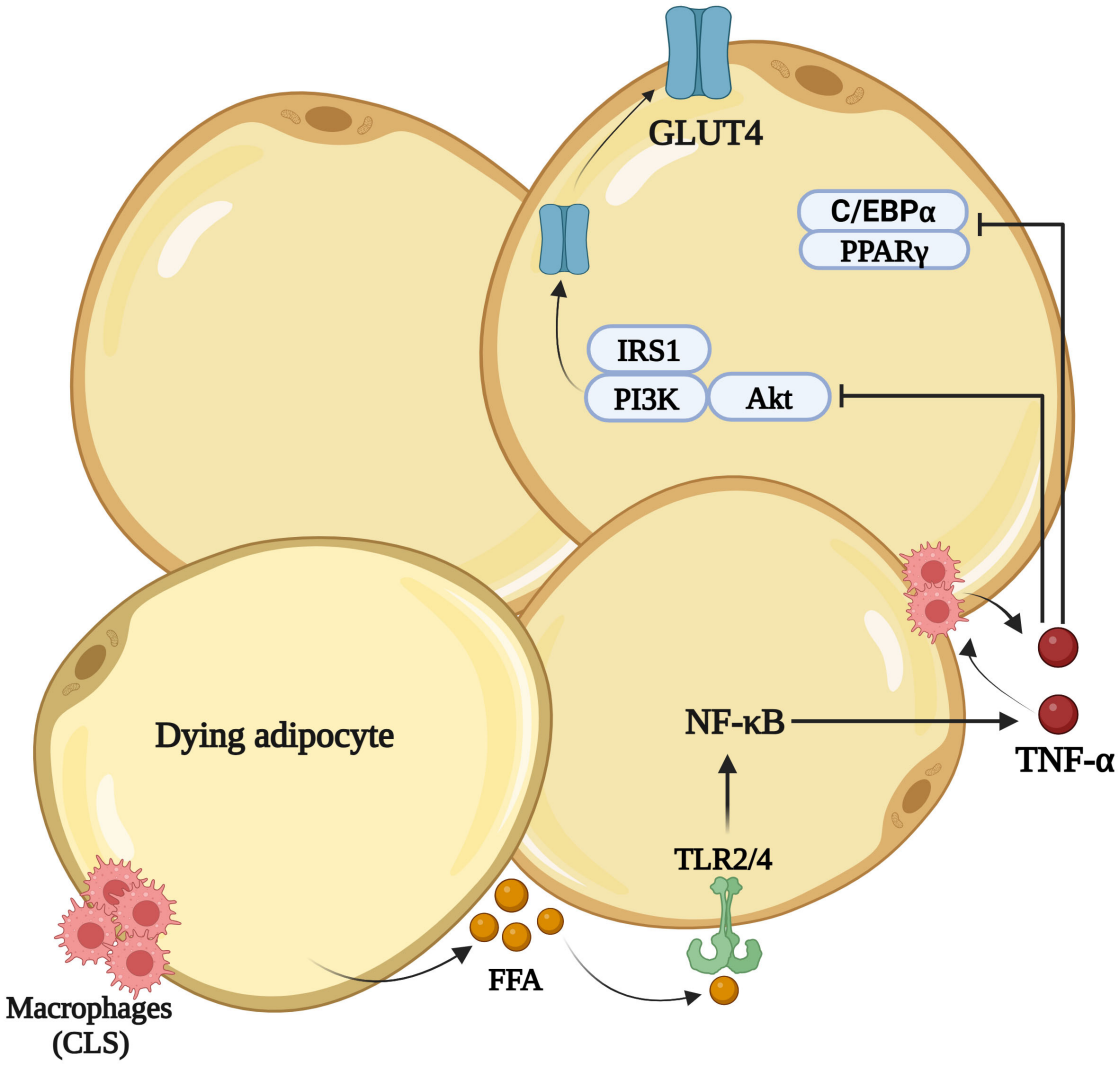
TNF-α induced Insulin Resistance: Unhealthy expansion of adipocytes during obesity causes release of free fatty acids (FFAs). This stimulates TNF-α secretion from adipocytes and macrophages which further encourages macrophages accumulation forming a crown like structure (CLS). TNF-α impairs the insulin response by inhibiting the phosphorylation of IRS-1, needed for GLUT4 (glucose transporter type 4) translocation. It downregulates the transcription of PPARγ and C/EBPα required for insulin responsiveness. Created in BioRender.

TNF-α is an inflammatory cytokine that influences metabolic responses primarily involved in IR (**Figure 2**). TNF-α is well known for its contribution to IR through its effect on GLUT4 and C/EBP expression within adipocytes (64). Additionally, it influences tyrosine kinase (TK) activity, thereby increasing the Ser/Thr phosphorylation of IRS-1, leading to IR (65). TNF-α is present not only in adipose tissue but also in skeletal muscle. About three decades ago, Hotamisligil et al. noticed upregulated TNF-α transcripts in the adipose tissue from four different genetically obese and diabetic rodent models (ob/ob, db/db, tub/tub and fa/fa) and neutralizing TNF-α improved insulin sensitivity and peripheral glucose uptake (11). Other studies also showed that weight loss and insulin sensitizing drugs reduced TNF-α and its receptor TNFR2 expression in the muscles of obese and diabetic mice (66).

Later several studies in humans confirmed these animal findings. Elevated TNF-α levels were observed in adipose tissue of Caucasian females with obesity and were positively correlated with plasma insulin and glucose levels (10). A study from Leiden reported increased TNF-α in serum of women with obesity (67). Similarly, other studies also consistently report increased levels of TNF-α in the serum or plasma of individuals with obesity (68–77). Expressions of TNF-α and its receptors TNFR1 and TNFR2 in the adipose tissue positively correlated with body mass index (BMI), adiposity, fat cell diameter, fasting plasma insulin and triglycerides in individuals with obesity (78–81). Weight loss reduced the levels of plasma TNF-α in females with obesity (82). Overall, elevated TNF-α levels in adipose tissue have also been reported across various ethnic groups (83–88) that correlated with BMI (68, 89), IL-6 (83) and macrophage markers (84). TNF-α and its receptors exhibit depot-specific differential expression (68, 85, 87, 89–91).

Interestingly, Tsigos et al. found that TNF-α levels were more strongly associated with impaired glucose tolerance than adiposity (91). In muscles, diabetic patients produce 4 times higher TNF-α than insulin-sensitive subjects (92). Research on Mexican and Danish population show higher TNF-α in insulin resistant and diabetic subjects (93, 94). Similarly, young individuals from the southern region of India with T2D demonstrate higher TNF-α levels (95). Elevated TNF-α in diabetic subjects from Gujarat (western India) is correlated with BMI, fasting blood glucose and plasma lipids (89). While among Pima Indians TNF-α is inversely related to fasting insulin (96) but positively associated with HbA1c levels—similar to findings in the Saudi population (96, 97). Insulin treatment in diabetic patients reduced inflammation, i.e., TNF-α and IL-6 (98). A large scale Potsdam study of 27,000 participants showed increased TNF-α in T2D but was not an independent predictor for T2D (99). However in another study by Ingelsson et al. on aged cohorts (70 years) from Uppsala community, TNF-α was found to be independently associated with metabolic syndrome (100). In African American women no difference in TNF-α between diabetic and non-diabetics was noted (101) (**Table 1**). Similarly, a study on post-menopausal women (Caucasians and African Americans) found no significant difference in TNF-α levels between individuals with or without obesity. This study also reported weight loss did not change TNF-α levels (102). Similar results were also observed in other reports (103–108). Overall, TNF-α is now accepted as a key inflammatory cytokine associated with obesity and IR. Future studies should focus on its prognostic value and tissue-specific roles to clarify whether it may serve as a predictive biomarker or therapeutic target in at-risk populations.

### 3.2 IL-6

**Figure 3:**
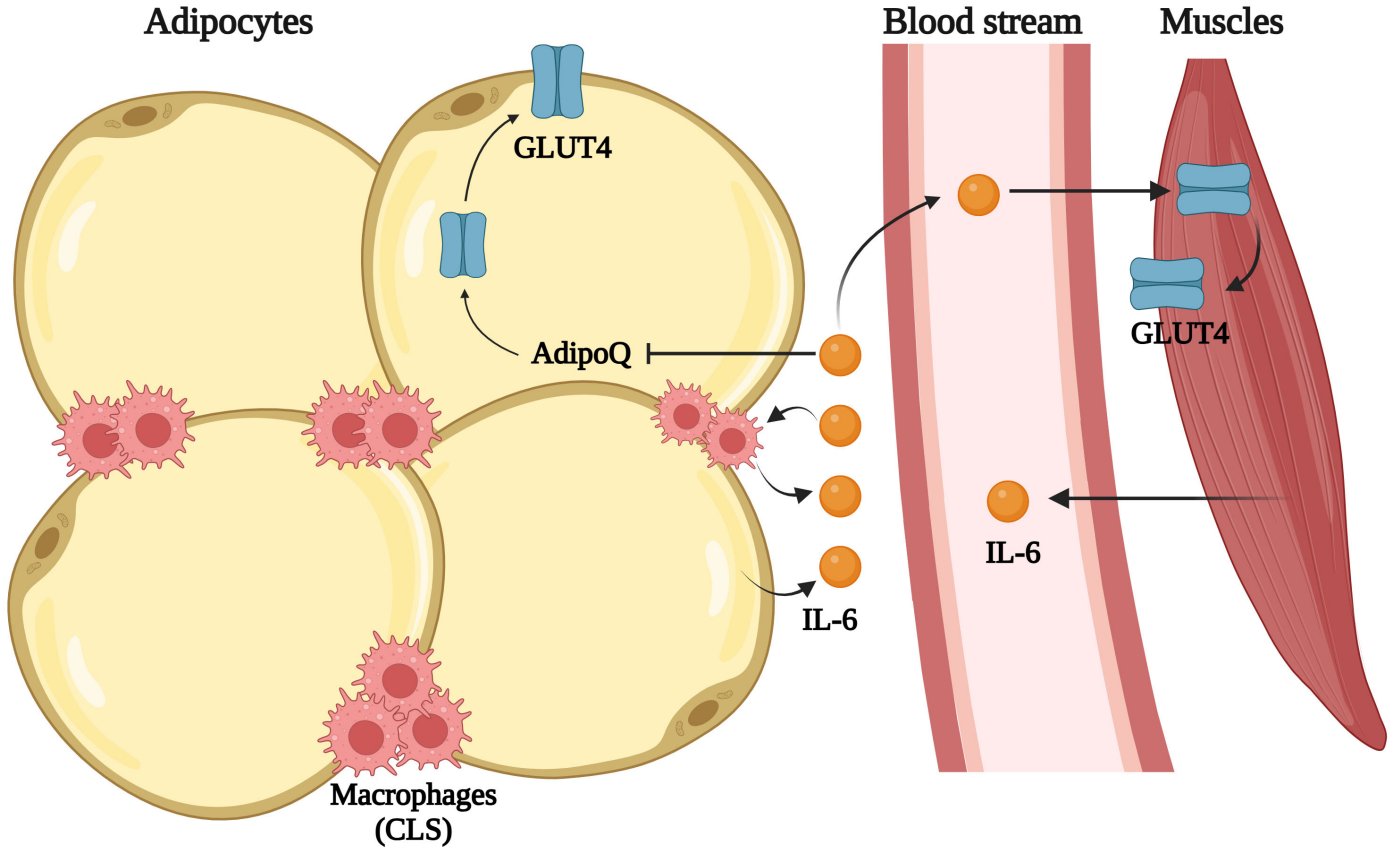
IL-6 effect on insulin sensitivity: Adipocytes and macrophages release IL-6 during obesity and reduce translocation of GLUT4 (glucose transporter type 4) on the cell surface, hindering glucose uptake. In muscle cells IL-6 promotes GLUT4 translocation. Created in BioRender.

IL-6 is known for its paradoxical role in obesity and IR (109). While it is often associated with promoting IR, IL-6 also enhances insulin sensitivity during physical activity. It is produced by adipocytes, macrophages, and T cells within the adipose tissue (110). Moreover, its role in macrophage polarization is unclear. Evidence from mouse models suggests IL-6 has both pro- and anti-inflammatory effects. Myeloid specific knockout of IL-6 increased adipose tissue inflammation and impaired glucose tolerance, indicating its anti-inflammatory properties while, deleting adipocyte-specific IL-6 showed reduced inflammation and macrophage accumulation (**Figure 3**) (111). In humans, it maintains glucose homeostasis in skeletal muscles and communicates with the adipose tissue to promote lipolysis (112, 113). Augmented IL-6 is noted in obesity and metabolic disease. Ali et al. showed that the release of IL-6 in subcutaneous adipose tissue (SAT) and blood is correlated with BMI in the Caucasian population (114). IL-6 is notably higher in visceral adipose tissue (VAT) than SAT (115–117). Elevated IL-6 expression is connected with BMI (68, 71, 83, 84, 86, 102, 117–124), TNF-α (83), and macrophage markers like CD163 (125) and MCP-1 (83, 125) in individuals with obesity. Weight loss decreases its circulating levels (102). Interestingly, there are two studies in 2020 with conflicting conclusions. Mikkawy et al. showed increased circulating levels of IL-6 and its positive correlation with BMI in Egyptians (126) whereas Mendez-Garcia et al. suggested a decrease in serum IL-6 as a potent marker of obesity and a negative correlation with BMI in Mexican population (69). Moreover, elevation in IL-6 levels is linked with weight loss in post laproscopic sleeve gastrectomy (127). IL-6 has been suggested as a key link between obesity and T2D (128). Furthermore, increased serum IL-6 concentration is reported in T2D Caucasian (94, 118, 129), Brazilian (108) and south Asian (India and Pakistan) populations (76). Circulating IL-6 levels correlate with fasting blood glucose (118) and HOMA-IR (75, 86). Potsdam study predicted IL-6 as an independent predictor of T2D that increases risk along with TNF-α and IL-1β (99). Furthermore, IL-6 is also linked to CRP (68, 96, 107, 130, 131), IL-4 (132), Resistin (133), IL-8 and MCP-1 (134), while inversely related to adiponectin (96, 130). Promoter polymorphism at IL-6, -174 G>C, is associated with metabolic syndrome (76). A report from the north Indian (Chandigarh) population showed increased IL-6 in individuals with obesity and T2D (130). However, studies from southern (Mysore) and northern (Jammu and Kashmir) regions of India did not find much difference in IL-6 levels between individuals with or without obesity (70, 105). There are other reports where no significant differences in serum levels of IL-6 between individuals with or without obesity or T2D was observed (100, 101, 103, 105). No difference in mRNA and serum levels in SAT and VAT was found (88, 135), and protein expression was diminished in SAT while elevated in VAT in individuals with obesity (135). Due to its fluctuating levels and context-dependent effects, longitudinal and tissue-specific studies are needed to determine its predictive reliability. In nutshell, IL-6 appears to be better correlated with obesity than T2D. Given its role in muscle energy metabolism, more clinical and mechanistic studies are required to understand its effect on T2D.

### 3.3 IL-1 family

IL-1 family of cytokines include IL-1α, IL-1β, IL-18, IL-33, IL-36α, IL-36β, IL-36γ, IL-36RA, IL-37 and IL-38. Some of these are important in pathogenesis of obesity and T2D (136). Obesity causes high concentrations of free fatty acid, which binds to TLR (2 and 4) to initiate inflammation and secretion of various cytokines, including the IL-1 family (137). IL-1β and IL-18 require cleavage by caspase-1 for their activation, which is regulated by inflammasome (138). High caspase-1 activity is reported in differentiated adipocytes and blocking it enhances the expression of PPARγ, GLUT4, and Adiponectin (139).

#### 3.3.1 IL-1β and IL-1α

IL-1β and IL-1α are not just secreted by immune cells in adipose tissue but also by adipocytes and regulate the pro-inflammatory cytokines (IL-17) and metabolism (140). IL-1β impairs insulin sensitivity by modulating PPARγ (141) mediated by caspase-1 (142). Treating obese mice with caspase-1 inhibitor shows reduction in IL-1β and improved insulin sensitivity (139). Similarly, ablation of IL-1Ra (interleukin-1 receptor antagonist) in mice reduced body weight and adipogenesis while increasing energy expenditure (143). Several studies have suggested increased plasma levels of IL-1β and IL-1α in individuals with obesity and T2D (124, 144). Some studies reported elevated IL-1α (100) but not IL-1β in individuals with obesity (103). Likewise, other studies observed no significant changes in IL-1 gene or protein expression in adipose tissue (88, 99, 135) or circulation (145). The role of IL-1α in obesity and T2D remains poorly defined. More targeted studies are needed.

As mentioned, IL-1β contributes mechanistically to IR. Treatment with an rIL-1 antagonist (anakinra) for 13 weeks improved glycemic control in T2D patients primarily by improving beta cell function and reducing inflammation (146). However, this study did not find any change in insulin sensitivity upon anakinra treatment. Another study showed that weight loss resulted in decrease in the systemic IL-1β levels and improved insulin sensitivity (147). Genetic variations in IL-1β gene and corresponding circulating levels are non-significantly associated with the risk of T2D (148). However, findings from India show regional variation. A study from western India (Gujarat) reported a four-fold increase in IL-1β expression among individuals with T2D (149), whereas a study from southern India found no significant difference in IL-1β levels between diabetic and non-diabetic groups (150). Thus, the IL-1β levels appears to be more relevant in islets compared to circulation or adipose tissue. IL-1β contributes mechanistically to IR, but we have inconsistent population-level data for its role in obesity.

#### 3.3.2 IL-18

IL-18 is produced by macrophages, dendritic cells, endothelial cells, Kupffer cells and vascular smooth muscle cells and is implicated in atherosclerosis (151). It regulates energy homeostasis by suppressing appetite and modulating fat distribution. It modulates metabolic homeostasis via activation of AMP Kinase and enhances lipid oxidation (152). IL-18 null mice demonstrated hyperphagia, increased adiposity and IR (153, 154). Anomalously, several studies have shown elevated IL-18 concentration in circulation and its increased expressions in the adipose tissues associated with BMI (145, 155). Increased plasma levels of IL-18 in individuals with obesity are also associated with IR, which is reduced after weight loss (156, 157). Although evidences are limited, current findings suggest that IL-18 is associated with both obesity and T2D and holds potential as a biomarker. However, comprehensive population-based studies are needed to clarify its precise role in the pathogenesis of these conditions.

#### 3.3.3 IL-36

The IL-36 cytokines belong to the IL-1 family and are key players in inflammatory diseases. The IL-36 subfamily includes three ligands—IL-36α, IL-36β and IL-36γ along with a natural antagonist, IL-36Ra (158). IL-36γ serum levels are elevated in individuals with obesity in comparison to lean individuals. Its serum levels correlate negatively and fasting blood glucose and HbA1c in individuals with obesity and T2D but not in those without T2D. This indicates its protective role against metabolic dysfunction during obesity (159). However, another study suggested that circulatory IL-36γ levels increase during obesity while reduce upon weight loss. Also, there is an increase in mRNA expression of both IL-36γ and IL-36R in VAT of obesity-associated T2D patients (160). Similarly, patients with T2D showed elevated expression of IL-36α and IL-36γ, accompanied by reduced expression of IL-36Ra (161). Children with obesity who are insulin-sensitive exhibit elevated serum levels of IL-36β, compared to insulin-resistant children, suggesting a protective role for IL-36 in metabolic health (162). The evidences so far suggest that IL-36 is elevated in obesity and may have protective effects on T2D. More research is required to clarify its mechanism.

#### 3.3.4 IL-33

IL-33 is structurally similar to IL-18, which signals via ST2 (Interleukin 1 receptor-like 1, or IL-1RL1) (163). It is constitutively expressed in endothelial cells in all the tissues and functions as “alarmin” in response to tissue damage or infection (164). Unlike other IL-1 family cytokines, IL-33 is inactivated upon cleavage by caspase-1 (164, 165). It is also expressed in various immune cells and non-immune cells including fibroblasts, myofibroblasts, osteoblasts, glial cells, adipocytes and smooth muscle cells. It is a potent pro-inflammatory mediator in diseases like parasitic infection, bacterial infection, fungal infection, asthma, arthritis, obesity and cardiovascular diseases (166, 167).

IL-33 has also been demonstrated to be protective against obesity. Administration of recombinant IL-33 in genetically obese and diabetic mice showed reduced adiposity, fasting glucose and improved insulin tolerance. This was accompanied by accumulation of M2 macrophages and higher Th2 cytokines (IL-10 and IL-13). Meanwhile, mice lacking ST2 (IL-33 receptor) had increased body fat and impaired insulin secretion and glucose regulation (168). A study by Wood et al. has found that in human IL-33 is mainly produced by both adipocytes and pre-adipocytes of omental white adipose tissues (169). Increased levels of IL-33 and ST2 expressions in adipose tissue of individuals with obesity is correlated with leptin levels (170). Similarly, in the Chinese population, circulating levels of IL-33 are elevated by obesity and positively correlated with metabolic disorders (171). In contrast, studies on Kuwaiti population have demonstrated diminished levels of IL-33 in individuals with normal weight with a negative correlation with BMI (172). IL-33 levels were also inversely correlated with HbA1c in normoglycemic and T2D patients. It was also associated with the genes involved in the beiging of the adipose tissue (173). Although the evidence is still emerging, IL-33 stands out for its protective effect on obesity. However, we cannot say anything definitive about its role in T2D.

### 3.4 IL-17

As discussed above, obesity promotes Th17 cells proliferation and thus increased secretion of IL-17. It inhibits adipocyte differentiation and promotes IL-6 production from adipocytes (174). Adipose stem cells and stromal vascular fraction derived from obese adipose tissue secrete IL-17. These cells suppress both adipogenesis and insulin responsiveness in adipocytes (175) by promoting the inflammatory microenvironment (176). Explants of omental fat from individuals with obesity secrete higher level of IL-17 in comparison to that from normal weight (140). However, another study reported no difference in IL-17 expression in VAT between individuals with or without obesity (135). IL-17 levels are elevated in circulation of T2D patients (161) as well as individuals with obesity (124, 177, 178). The data available so far establishes a role of IL-17 contributing to adipose inflammation and IR. Large population studies are required to confirm current interpretation.

### 3.5 IL-10

IL-10 is identified as an anti-inflammatory cytokine that signals via the JAK/STAT system. It promotes wound healing and maintains tissue homeostasis (179). It exhibits anti-inflammatory properties by inhibiting TNF-α secretion and promoting the release of TNFR (TNF receptor) from the surface of macrophages (180). It stimulates macrophages towards M2 or alternative phenotype and promotes eosinophil recruitment (181, 182). In macrophages, it promotes OXPHOS while inhibits glycolytic flux by suppressing mTOR activity (183). Mice over-expressing IL-10 in muscles showed less expression of MCP-1 (decreased macrophage infiltration), indicating its role in dampening inflammation (184). High glucose levels impair IL-10 signaling by blocking STAT3 activation, limiting its ability to inhibit TNF-α secretion in macrophages (185).

A Leiden study on elderly concluded that low IL-10 production is associated with T2D and metabolic syndrome (186). Similarly, among African American women decreased levels of IL-10 is observed with increase in weight (121). Decreased IL-10 levels are correlated with triglyceride levels in the Mexican (69, 93) and Chinese population (187). Similarly, IL-10 is negatively associated with BMI in Japanese women (61). Likewise, a diminished amount of IL-10 is also correlated with T2D and HOMA-IR in different ethnic population (123, 131, 188). On the contrary, several studies showed increased levels of IL-10 in individuals with central obesity (71, 72, 103, 124), but not in general obesity (104). High levels of IL-10 correlated with metabolic syndrome among individuals with obesity (72) and T2D (144). In parallel, increased IL-10 is associated with metabolic syndrome, HOMA-IR and glucose infusion rate in south Indians and Caucasians (84, 118, 150). However, several studies showed no significant differences in IL-10 levels between individuals with or without obesity (67, 100, 108, 132, 135). Although IL-10 is an established anti-inflammatory cytokine, population data provide inconsistent findings, making it difficult to definitively determine its role in obesity and T2D.

### 3.6 IL-4 and IL-13

IL-4 and IL-13 are anti-inflammatory cytokines, similar to IL-10, and stimulate macrophages towards alternative activation via distinct pathways (189). IL-13 is known to be involved in glucose homeostasis. Stanya et al. revealed that IL-13 affects glucose tolerance and insulin sensitivity (190). Diminished levels of IL-13 in serum and myotubes from T2D patients indicate its importance in glucose metabolism in skeletal muscle cells (191). Similarly, IL-13 plasma levels are higher in individuals with obesity (104) and insulin-resistance (93).

IL-4 suppresses adipogenesis in 3T3-L1 through STAT6 (192) and modulates the transition between glucose metabolism and fatty acid oxidation (FAO) in hepatocytes by repressing PPARα via STAT6 (193). Elevated IL-4 is found to be associated with BMI independent of T2D in African American women (101). Both IL-13 and IL-4 are found to be elevated in T2D (71, 144) and metabolic syndrome (150, 171) and correlated with fasting blood glucose (150). However, other studies show no distinction in the circulating amount of IL-13 and IL-4 among individuals with or without obesity or T2D (67, 103, 132, 135). The available data broadly suggests that these cytokines may be higher in insulin-resistant individuals but more data is required to say anything with certainty, especially for obesity.

### 3.7 IL-2

IL-2 is a pro-inflammatory cytokine, primarily produced by T cells (194). IL-2 secretion from adipose tissue-resident iNKT cells regulate the anti-inflammatory phenotype of macrophages (195). Elevated expression of IL2 have been reported in the adipose tissue of individuals with obesity. It is correlated with macrophage marker (CD163), cytokines (IL-8 and IL-12a), chemokines (CCL2 and CCR5) as well as clinical parameters like BMI, fasting blood glucose and blood triglyceride levels supporting its potential as an obesity marker (196). Serum IL-2 is also increased in obesity (71, 124) and metabolic syndrome, showing a positive correlation with fasting blood glucose and C-reactive protein (CRP) while negative correlation with adiponectin (150). It is associated with T2D independent of BMI in African American women (101). On the other hand, many studies demonstrated no alterations in serum concentration (67, 100, 103, 104, 132) or decreased levels in the circulation with obesity or T2D (197). IL-2 levels are variably altered in obesity and may reflect immune status rather than serve as a direct biomarker of metabolic disease. However, with the limited data we can say it is better correlated with T2D.

### 3.8 IL-15

IL-15 is a pleiotropic cytokine structurally similar to IL-2. It is expressed in many immune cells like T cells, macrophages, DCs, mast cells and non-immune cells like keratinocytes, epidermal skin cells, fibroblasts, epithelial cells, skeletal muscle and adipocytes (198). IL-15 stimulates glucose uptake in skeletal muscle (199) by facilitating GLUT4 translocation via JAK3/STAT3 pathway (200). Its over-expression in muscle and increased levels in circulation reduces adiposity (201, 202). Animal studies suggest that IL-15 improves, insulin sensitivity, promotes weight loss by upregulating lipid oxidation genes and improvs glucose homeostasis (203, 204). IL-15 treatment affects adipocyte lipid accumulation mediated by altered mitochondrial activity (205). Various studies have shown decreased expressions of IL-15 in individuals with obesity and T2D (202, 206, 207). Similarly, plasma levels of IL-15 are also negatively correlated with BMI (206). However, a study by Zalm et al. found increased levels of IL-15 in individuals with obesity and metabolic syndrome (124). Another report suggests no difference in IL-15 serum levels in individuals with either conditions (135). Although the mechanistic studies find IL-15 to be beneficial in both obesity and T2D, the clinical data only supports its role in T2D and not obesity. Larger population studies might be required to establish its role unequivocally.

### 3.9 TGF-β

TGF-β (transforming growth factor-beta) family includes pleiotropic cytokines regulating various biological processes involving fibrosis development and tissue homeostasis. The signal transduction is mediated via Smad phosphorylation (208). TGF-β-Smad3 pathway has an important contribution in the pathogenesis of obesity and T2D (209). It enhances the release of proinflammatory cytokines (IL-1β, TNF-α and IL-6) and thus reduces leptin expression in 3T3-L1 derived adipocytes (210). Moreover, TGF-β impairs adipocyte catabolism independent of inflammation (140). In line with these findings, higher TGF-β and TNF-α are reported from WAT in obese mice (211). In a mice model, deletion of Smad3 protects against obesity and IR, with increased mitochondrial biogenesis in adipocytes (212).

In line with this, TGF-β release is enhanced during obesity in human adipose tissue (213, 214) and circulation (212, 215). It is also associated with BMI (214), adiposity and VO_2_ consumption while inversely related to HOMA-IR (212). Studies have shown significantly elevated levels of TGF-β in T2D patients (216, 217). Higher amounts of TGF-β in patients with diabetic retinopathy and nephropathy are correlated with HbA1c (218). T29C polymorphism in the signal peptide sequence region of TGF-β1 contributes to the prevalence of obesity and T2D (219). Although population studies are limited, the current evidences suggest that TGF-β is associated with obesity as well as T2D.

### 3.10 Adiponectin

**Figure 4:**
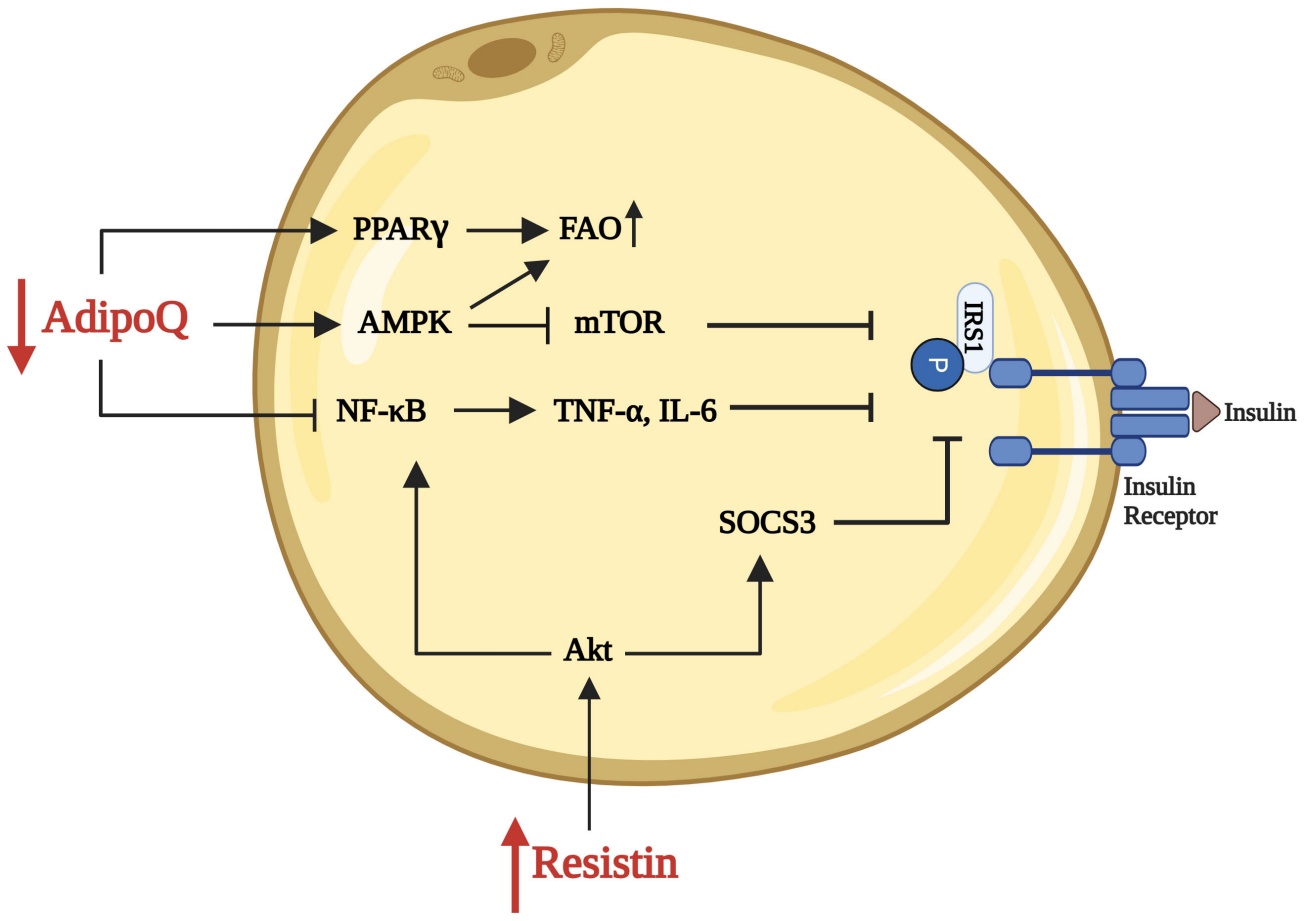
Resistin and adiponectin in adipose tissue affecting insulin signaling during obesity: Increased levels of resistin, associated with obesity, activate the NF-κB pathway, which subsequently increases the expression of pro-inflammatory cytokines, including TNF-α and IL-6. These cytokines activate SOCS3, which inhibits the phosphorylation of IRS-1, a key molecule in the insulin signaling pathway. This inhibition prevents downstream activation of Akt, thereby impairing glucose uptake and further promoting insulin resistance in adipose tissue. Reduced levels of adiponectin (AdipoQ) impair the activation of PPARγ and AMPK pathways, which normally promote fatty acid oxidation (FAO) and inhibit mTOR respectively. This impairment leads to decreased FAO and altered cellular metabolism, contributing to insulin resistance. Created in BioRender.

Adiponectin is an adipokine predominantly secreted by adipocytes. It was first reported in 1995 as Acrp30 (Adipocyte complement-related protein of 30kD, due to its structural resemblance to complement factor C1q) in differentiated 3T3-L1 adipocytes having some role in homeostatic control of metabolism (**Figure 4**) (220). Simultaneously, other groups independently described it as apM1 (Adipose Most Abundant Gene Transcript1) (221), GBP28 (Gelatin Binding Protein of 28kDa) (222) and AdipoQ secreted exclusively by adipocytes (223). Adiponectin enhances glucose uptake and protects from IR mediated via AMPK. It also suppresses inflammation by inhibiting NF-κB pathway (224, 225). Hu et al. pointed out that AdipoQ is similar to Acrp30, and its expression is significantly depleted in adipose tissues of obese mice, rats and humans (223). In 1999, Arita et al. showed reduced plasma adiponectin concentration and its negative correlation with BMI in the Japanese population (226). This study also reported males exhibiting lower levels than females across different weight groups (226). This pattern was later confirmed by several other population-based studies (70, 106, 227–230).

Consistently, lower circulating adiponectin levels have been documented in individuals with obesity (70, 72, 130, 197, 207, 231) and T2D (95, 96, 130, 228, 230–232). Studies from different regions of India have also shown the inverse relationship of adiponectin with insulin sensitivity and CRP (discussed below) while positive correlation with IL-6 (96, 130, 230). However, contrasting results have also been found where there is no significant difference in serum concentration (105) and mRNA expressions in adipose tissues from individuals with or without obesity or T2D (88). Overall, majority of evidences indicate that adiponectin levels are reduced in both obesity and T2D, highlighting its potential protective role against IR. Given its consistent correlation with metabolic health, it holds considerable promise as a diagnostic biomarker.

### 3.11 Leptin

In 1994, Leptin was discovered as a peptide hormone produced by the adipose tissue, which directs food intake and energy expenditure (233). Subsequently, it was demonstrated that administration of recombinant leptin in genetically obese mice reduces adiposity, increases energy expenditure and reduces hyperphagia (234, 235). In humans, however, there is decreased sensitivity to endogenous leptin in individuals with obesity (236). Homozygous mutation in leptin receptor (LEPR) results in early onset of obesity (due to hyperleptinemia) and this leads to several endocrine abnormalities including hyperinsulinemia (237). Its expression in the adipose tissue and levels in serum are significantly elevated in individuals with obesity (67, 74, 95, 129, 131, 236) which is improved by weight loss (74, 131, 236). Higher leptin in circulation and adipose tissue correlates well with BMI in different ethnic populations (87, 95, 106, 131, 177, 236, 238, 239). Studies have also pointed out that women secrete more leptin than men (106, 238) and it is expressed more in SAT than in VAT (87, 88, 90). Leptin concentration in the portal vein is 20% less than in the peripheral artery (240). Leptin is involved in glucose regulation (241) and reduced plasma level of leptin is associated with IR and T2D (241–243). Serum leptin concentrations are significantly elevated in individuals with obesity and T2D compared to those without obesity, demonstrating that circulating levels are primarily BMI-dependent (244). In newly diagnosed T2D patients, Metformin/Sitagliptin therapy lowers serum leptin levels and improves lipid profile (245). It is also associated with other factors related to obesity like inflammatory markers IL-6 (125), CRP (106, 125, 131), IL-33 (170) and metabolic markers like HOMA-IR, HbA1c (95, 129) and fat content (236, 238). Yet, it displays no link with increased levels of IL-17 and IL-23 in obese women (177). Overall, leptin levels increase with adiposity and when this relationship is broken, also known as leptin resistance, it leads to IR and other metabolic disturbances (241, 246). However, unlike insulin, external leptin injection failed to improve T2D (247).

### 3.12 Resistin

Resistin is a cytokine produced by adipocytes as well as several immune cells. High levels are noted in mice during obesity, leading to IR and thus called Resistin, “for resistance to insulin” (248). Resistin modulates β-oxidation of fatty acids by down-regulating the PGC-1α. Furthermore, PPARγ is well known for suppressing the resistin levels (**Figure 4**). Macrophages are the main immune cell which secrete resistin and affect obesity and related cardiovascular complications (248–250). Human macrophage-derived resistin provoked mice’s adipose tissue inflammation and IR (251).

In humans, elevated resistin levels have been linked to reduced insulin sensitivity in individuals with obesity (Hispanic population) (252). Increased resistin expression has been found in SAT from individuals with obesity compared to those without and its circulating levels are positively correlated with triglycerides and HDL (207). Similarly, elevated resistin in circulation was noticed in newly diagnosed T2D women with coronary heart disease (133, 253). In a study by Stepien et al., waist-to-hip ratio showed a negative association with resistin levels in individuals with obesity without gender differences (106). Among children and adolescents, however, it is not associated with obesity but influenced by pubertal age, with higher levels in girls than boys (254). Resistin is a pro-inflammatory adipokine elevated in obesity and T2D, contributing to IR but needs more validation in human cohorts.

### 3.13 Chemokines

These belong to the chemotactic family of cytokines involved in the trafficking and migration of immune cells (like macrophages) and thus, critical components in inflammation. Plata-Salaman et al. suggested that microinfusion of CCL2 (aka MCP-1) and CCL5 (aka RANTES) reduced short-term food intake in Wistar rats (255). CCL2 is considered a crucial chemokine in obesity and T2D. Mice having a deletion in CCL2 or its receptor CCR2 attenuate ATM accumulation and enhance insulin sensitivity when fed a high-fat diet (256). CCL2 promotor SNP is also associated with diabetic nephropathy (257). Accumulation of CCR5^+^ macrophages occurs during obesity and CCL5 expressions are upregulated in the adipose tissue. Deletion of CCR5 reduces macrophage recruitment, shifts macrophage phenotype towards M2 and improves glucose homeostasis (258). However, the chemokine’s effect on macrophage polarization is not well understood mechanistically.

Several studies reported increased chemokines in both adipose tissue and circulation during obesity. Huber et al. analyzed the alterations in the levels of chemokines in individuals with obesity (122) and reported higher levels of CCL2 and CCL5. Increased CCL2 in circulation and adipose tissue have been consistently reported in individuals with obesity across different ethnic groups including Danish, Korean and Polish populations (125, 134, 155). CCL2 expression in the adipose tissue showed a positive association with BMI and markers of macrophages (CD11b, CD163, CD206, CD14, CD68) (83, 84) along with TNF-α (83) and IL-6 (131). Comparable findings were reported in the Korean population (134). In the Iranian population however, CCL2 levels were inversely associated with waist circumference (103). Huber et al. also observed elevated mRNA expression of several chemokines and their receptors in adipose tissue—specifically CCL3 (MIP1α), CCL5, CCL18, CCL11 along with CCR1, CCR2, CCR3 and CCR5. However, among these, only CCL5 demonstrated a significant increase in plasma concentration in individuals with obesity compared to healthy weight controls. CCL2, CCL3 and CCL8 mRNA expressions were elevated in SAT compared to VAT, while higher CCL11 level was seen in VAT. CCL11 shows a positive correlation with macrophage infiltration (122). CCL3 levels as well are higher in individuals with obesity in circulation (124) and VAT (88) compared to healthy weight individuals. CXCL16, CCL16 and CCL2 are associated with T2D independent of BMI in African American women (101). Additional correlations include an inverse relationship between CCL7 and IL-33 in Kuwaiti population (172). Along similar lines, CCL5, CCL19 and CCR2 have been correlated with IL-2 in individuals with obesity (196). However, some studies suggested no significant change in chemokines during obesity (103, 135). The majority of evidence supports increased levels of CCL2 and CCL5, reinforcing their roles in adipose tissue inflammation and obesity but not T2D. For other chemokines, there are very few clinical reports.

#### 3.13.1 IL-8

IL-8, a chemokine (CXCL8), is highly produced in adipose tissues during obesity (259). It contributes to IR by inhibiting Akt phosphorylation (260). Increased circulating levels of IL-8 are reported in individuals with obesity, including children (71, 124, 259). In the obese Korean population, it is found to be associated with IL-6 and CRP besides BMI (134). Bruun et al. showed higher IL-8 production in VAT than in SAT (116). Its mRNA expression in VAT and SAT correlated with BMI and IL-2 (88, 196). It is also noted higher in T2D patients (144). Some other reports suggested no significant difference between individuals with or without obesity or metabolic syndrome (100, 103, 117). While, present data is suggestive of role of IL-8 in obesity and T2D, further research is needed to validate its utility as a reliable biomarker for T2D.

### 3.14 C-reactive protein

CRP is a marker of systemic inflammation. Visser et al. proposed that obesity is associated with low-grade systemic inflammation by showing its augmented levels in obesity and association with BMI (261). A study on a middle-aged population consisting of 95% Caucasians showed a positive correlation with BMI regardless of metabolic syndrome in individuals with obesity (262). Elevated CRP has been reported in individuals with obesity from several ethnic groups including Indians, Koreans, Japanese, Germans, Pima Indians, Finnish and Mexicans (67, 68, 74, 99, 107, 122, 123, 129, 130, 134, 145). Increased CRP levels are also reported in T2D and metabolic syndrome patients (100, 130, 144, 206, 229, 263), whether men or women. In an interesting investigation by Forouhi et al., South Asian women have been found to have higher visceral fat and median CRP levels than European women, though in both groups CRP is positively linked with insulin and lipid levels (264). Therefore, it is well correlated with BMI (75, 107, 118, 134) and decreases with weight loss (102). Similarly, it is linked to fasting blood glucose and HOMA-IR (94, 108, 118, 263) and is considered a predictor of T2D (119, 265, 266). Elevated CRP concentrations are further associated with an increased risk of diabetic nephropathy and cardiovascular disease (CVD) in patients with T2D (267, 268). Besides, it is positively associated with IL-8 (134, 145), IL-6 (96, 118), resistin (133) and MCP1 (134) while negatively related to adiponectin (96, 197) and IL-2 (197). However, not all findings are consistent—some populations, such as Caucasians (135) and South Indians (70) with obesity did not show significant differences in CRP levels compared to healthy weight individuals. Similarly, some studies reported no relation of CRP with IR (106, 125, 173).

Overall, CRP is a non-specific but consistent marker of systemic inflammation in obesity and T2D. Though not mechanistically causal, it may support diagnosis and risk prediction when interpreted alongside other markers.

## 4 Discussion

Cytokines are central inflammatory mediators within the inflammatory model of obesity and T2D, linking metabolic dysfunction to immune system dysregulation. This review critically examines whether cytokine profiles observed at the population level consistently reflect their association with obesity and T2D.

Our collection of over 100 clinical studies across diverse cohorts reveals that several cytokines—such as TNF-α, IL-6, CRP, leptin, resistin and adiponectin—are repeatedly altered in both obesity and T2D. However, substantial variability in cytokine levels persists and their levels vary significantly depending on ethnic, regional and methodological factors.

Although a wide range of cytokines has been found to correlate with metabolic parameters such as BMI, IR and HbA1c, the current literature is dominated by cross-sectional studies and most studies do not establish causation and very few explore predictive potential. For example, TNF-α and IL-6 levels are consistently elevated in both obesity and T2D across numerous cohorts (**Table 1**), yet limited longitudinal data exist to demonstrate whether these cytokines rise prior to disease onset or actively contribute to disease progression. Similarly, adiponectin consistently shows a protective profile but its diagnostic value remains unclear in prospective studies (**Table 1**). These observations underscore a crucial distinction: while cytokine alterations are clearly associated with metabolic dysregulation, whether they act as drivers, consequences, or passive markers of disease remains unresolved in most cases. Although individual cytokines show promise, their standalone diagnostic use is unreliable due to overlap with other inflammatory conditions and inter-individual variability. A combined cytokine panel may offer improved diagnostic value. Based on consistency across populations, a panel including TNF-α, leptin, resistin, adiponectin and IL-6 could be considered. However, cytokines like CRP despite being a general marker of inflammation, should not be ignored as it may still carry diagnostic utility in the absence of infection.

One major challenge in interpreting cytokine data lies in the heterogeneity of metabolic responses among individuals with obesity. A subset of individuals, often termed “metabolically healthy obese” (MHO), exhibit elevated BMI but maintain normal insulin sensitivity, lipid profiles and blood pressure. Intriguingly, the cytokine profiles of MHO individuals often resemble those of lean, metabolically healthy controls, suggesting that the presence of excess adipose tissue alone does not inevitably drive a pro-inflammatory state (269). Despite their healthier metabolic profiles, MHO individuals are not immune to disease progression. Several studies suggested that MHO status may be transient, with a significant proportion transitioning into metabolically unhealthy obese (MUO) over time. The role of lifestyle interventions such as regular exercise, dietary modifications and weight management in maintaining MHO status remains critical. This finding challenges the assumption of a linear relationship between adiposity and inflammation and underscores the need to consider immune cell phenotypes (e.g., M1 vs. M2 macrophages), tissue-specific inflammation and adaptive immune responses (e.g., Treg/Th17 cell balance) when evaluating cytokine signatures. The existence of the MHO phenotype highlights the importance of immunometabolic context and suggests that inflammation arises from more than just adipose expansion (269, 270).

Despite significant advances, current cytokine research in metabolic diseases faces several persistent limitations. One, a major issue is the predominance of cross-sectional studies, which provide only a snapshot of cytokine levels and limit our ability to assess temporal dynamics or infer causality. Second, methodological inconsistencies—such as differences in assay types, sample processing and measurement platforms—further complicate comparisons across studies. Third, key populations such as children and adolescents are underrepresented, restricting our understanding of cytokine trajectories from early life or pre-disease stages. Fourth, ethnic and regional variability is also often overlooked, despite its significant influence on both metabolic risk and cytokine expression. These shortcomings make it difficult to determine whether cytokine alterations serve as early indicators or simply reflect ongoing metabolic dysfunction.

To realize the full potential of cytokines as diagnostic and prognostic biomarkers in obesity and T2D, future research should prioritize the longitudinal cohort studies, multi-ethnic-diverse populations and integration with lifestyle and intervention data. Ultimately, integrating cytokine profiling with genetic, metabolic, and clinical parameters may enhance early diagnosis and individualized risk stratification in metabolic diseases.

## 5 Conclusion

Obesity and T2D are characterized by a chronic inflammatory state, in which cytokines and adipokines play central roles. This review assessed whether cytokine profiles consistently reflect this inflammatory model across populations and whether these profiles have diagnostic or predictive value. Current evidence supports the elevation of several pro-inflammatory cytokines—such as TNF-α, IL-6, CRP, leptin, and resistin—in both obesity and T2D. Conversely, protective or anti-inflammatory cytokines like adiponectin and IL-33 are often reduced or functionally distinct in these conditions. However, significant variability exists across studies in terms of cytokine measurement methods, population demographics, disease stage and co-morbidities. This limits the ability to draw firm conclusions about causality or prediction. We conclude that while cytokines do conform to the inflammation model of obesity and T2D, their clinical utility as standalone predictive or diagnostic markers is limited. A multi-cytokine panel—potentially including TNF-α, leptin, resistin, adiponectin and IL-6—may offer a more robust approach, but this requires validation through large-scale, prospective studies. CRP, although commonly elevated, should not be excluded from diagnostic panels solely based on its role in infection; its elevation in non-infectious metabolic inflammation warrants further exploration. Future studies should focus on time point studies from diverse populations and integrated approaches that link cytokine data with clinical, genetic and lifestyle factors to clarify how inflammation contributes to obesity and T2D development.
